# Prognostic significance of early systolic blood pressure variability after endovascular thrombectomy and intravenous thrombolysis in acute ischemic stroke: A systematic review and meta‐analysis

**DOI:** 10.1002/brb3.1898

**Published:** 2020-10-14

**Authors:** Jingcui Qin, Zhijun Zhang

**Affiliations:** ^1^ Department of Neurology Affiliated ZhongDa Hospital School of Medicine Research Institution of Neuropsychiatry Southeast University Nanjing China; ^2^ Department of Neurology Xuzhou First People's Hospital, The Affiliated Hospital of China University of Mining and Technology The Affiliated Xuzhou Municipal Hospital of Xuzhou Medical University Xuzhou China

**Keywords:** blood pressure, endovascular recanalization, ischemic stroke, meta‐analysis

## Abstract

**Objectives:**

Previous studies have shown inconsistent results regarding the effect of early systolic blood pressure variability (SBPV) after endovascular thrombectomy (EVT) and intravenous thrombolysis (IVT) on functional outcome in acute ischemic stroke (AIS). The **s**ystematic review and meta‐analysis aimed to determine the effect of early SBPV after EVT and IVT on outcome in AIS.

**Materials and Methods:**

We searched for articles published before February 2020 in the following databases: PubMed, Web of Science, EMBASE, Medline, and Google Scholar. The pooled multivariate odds ratios (ORs) or relative risks (RRs) and 95% confidence intervals (CIs) were obtained using STATA 13.0 software.

**Results:**

Increased early SBPV after EVT was significantly associated with worse functional outcome in AIS (OR = 1.42, 95% CI 1.02 to 1.99, *I* ^2^ = 82.4%, *p* value of *Q* test < .001), whereas no significant associations were indicated between SBPV after IVT and functional outcome, symptomatic intracerebral hemorrhage (sICH) in AIS [functional outcome: RR = 1.08, 95% CI 0.96 to 1.22, *I* ^2^ = 0.0%, *p* value of *Q* test = 0.793; sICH: RR = 2.40, 95% CI 0.71 to 8.03, *I* ^2^ = 78.2%, *p* value of *Q* test = 0.01].

**Conclusions:**

The present study provided evidence that increased early SBPV after EVT is related to worse longer‐term functional outcome in AIS, but the association is not significant in AIS patients treated with IVT. Furthermore, individualized BP management strategies were essential for AIS patients after EVT or IVT.

## Graphical abstract

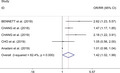

## 1 INTRODUCTION

Previous studies indicated that functional outcome after ischemic stroke (IS) is influenced by some factors including stroke severity, age, initial glucose, and time to and success of recanalization (Jauch et al., 2013; Powers et al., 2015). Systolic blood pressure variability (SBPV), independent of mean absolute BP level, is also an important factor to the outcome of acute ischemic stroke (AIS) (Rothwell, 2010; Rothwell et al., 2010). However, controversial results have been obtained from studies regarding BP management and its influence on functional outcome (Bennett et al., 2018). A recent systematic review and meta‐analysis showed that increased BPV in AIS might be related to worse functional outcome (Manning et al., 2015). Two studies indicated that worse outcomes with increased BPV might be attributed to increases in infarct volume (Delgado‐Mederos et al., 2008; Endo et al., 2013). Cerebral blood flow reperfusion, the clinical goal of endovascular thrombectomy (EVT) and intravenous thrombolysis (IVT), is closely associated with better prognosis in AIS (Coutinho et al., 2017; Fjetland et al., 2015). Regarding the effect of early BPV after EVT and IVT on functional outcome in AIS, previous studies showed inconsistent results. Bennett et al. (2018) indicated that increased BPV after EVT predicts worse neurologic outcomes in patients with AIS. However, some studies showed no significant associations between early BPV after EVT (Cho & Kim, 2019) or IVT (Tomii et al., 2011) and functional outcome. The study aimed to determine the effect of early SBPV after EVT and IVT on outcome in AIS by undertaking a **s**ystematic review and meta‐analysis.

## 2 METHODS

The present study was performed based on the Preferred Reporting Items for Systematic reviews and Meta‐Analysis (PRISMA) guideline (Moher et al., 2009).

### 2.1 Search strategy and selection criteria

In the study, articles published before February 2020 were searched in the following databases: PubMed, Web of Science, EMBASE, Medline, and Google Scholar. Search terms were as follows: (‘stroke’) AND (‘blood pressure’) AND (‘thrombectomy’ OR ‘thrombolysis’). After eliminating duplicates, 634 studies were included. After reading the full‐text articles by two independent researchers, studies were included if they report odds ratios (ORs) or relative risks (RRs) and 95% confidence intervals (CIs) for the effect of a defined increment (eg, per 10‐mm Hg increase or 1‐standard deviation (*SD*) increase) in either early (less than 48 hr after EVT or IVT) *SD* of SBP or coefficient of variation (CV) of SBP on outcome after EVT or IVT in AIS. Those studies were also included if the ORs and 95%CI could be calculated from the data provided in the studies. Studies were excluded if they included AIS patients receiving IVT before EVT. Additionally, meta‐analyses, reviews, and case reports were eliminated from the study.

### 2.2 Data extraction

We extracted data as follows: Author, publication year, study design, study location, sample sizes, information of participants (age, average initial stroke severity, pretreatment systolic blood pressure), therapy given, antihypertensive treatment, time from stroke onset to recruitment, BP measurement technique, and duration of monitoring, blood pressure variability (BPV) parameters included in analyses and outcome measures.

### 2.3 Meta‐analysis

In the present study, STATA 13.0 software was used. *Q* test and *I* ^2^ were used to explore the heterogeneities between studies. Random effects models were applied as pooling methods with invariably high heterogeneity (*p* value for *Q* test ≤ .05); fixed effects models were used with invariably low heterogeneity (*p* value for *Q* test >.05). In addition, sensitivity analysis was applied to evaluate the stabilization of the study. Begg's test, Egger's test, and funnel plot were applied to assess publication bias. Quality appraisal was performed with the Cochrane Risk of Bias Tool. Data were analyzed with Review Manager 5.3.

### 2.4 Ethical statement

The present study was a meta‐analysis. No ethical statement is provided.

## 3 RESULTS

### 3.1 Study selection and characteristics

**FIGURE 1:**
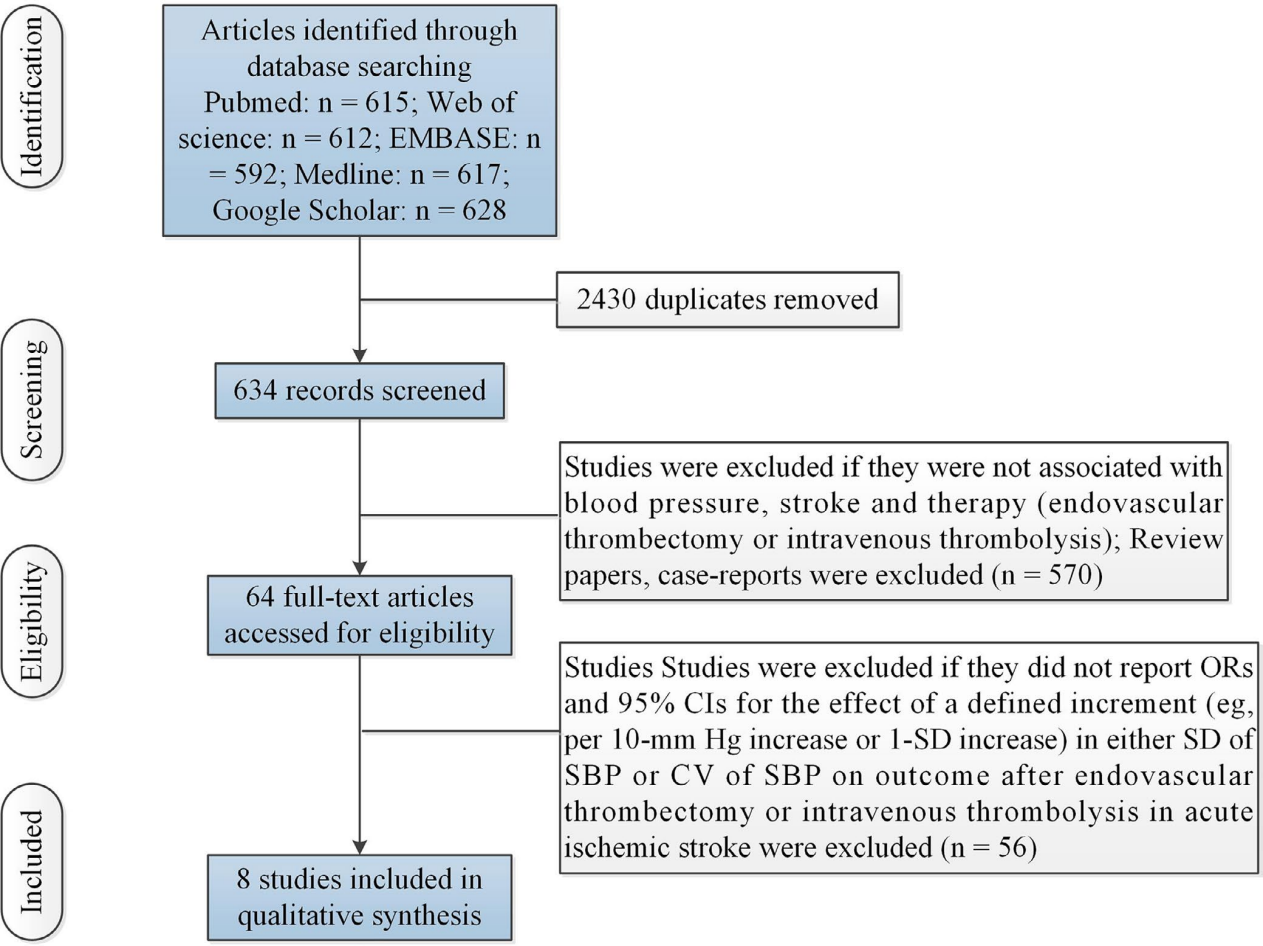
Flow of information through the different phases of the meta‐analysis. Abbreviations: CI, confidence intervals; CV, coefficient of variation; EVT, endovascular thrombectomy; IVT, intravenous thrombolysis; OR, odds ratio; SBP, systolic blood pressure; *SD*, standard deviation

Figure 1 illustrated the selection procedures. Table S1 showed study characteristics of included studies. In these studies, five studies (Anadani et al., 2019; Bennett et al., 2018; Chang et al., 2018, 2019; Cho & Kim, 2019) (including 2198 AIS patients) investigated the effect of a defined increment (per 10‐mm Hg increase or 1‐*SD* increase) in early *SD* or CV of SBP on functional outcome after EVT in AIS. Among the five studies, two studies (Anadani et al., 2019; Cho & Kim, 2019) explored the associations between SBPV and symptomatic intracerebral hemorrhage (sICH) or mortality after EVT in AIS. Three studies (Berge et al., 2015; Endo et al., 2013; Tomii et al., 2011) (including 3687 AIS patients) explored the effect of a defined increment (per 10‐mm Hg increase or 1‐*SD* increase) in early CV or *SD* of SBP on functional outcome and sICH after IVT in AIS.

### 3.2 Meta‐analysis results

**FIGURE 2:**
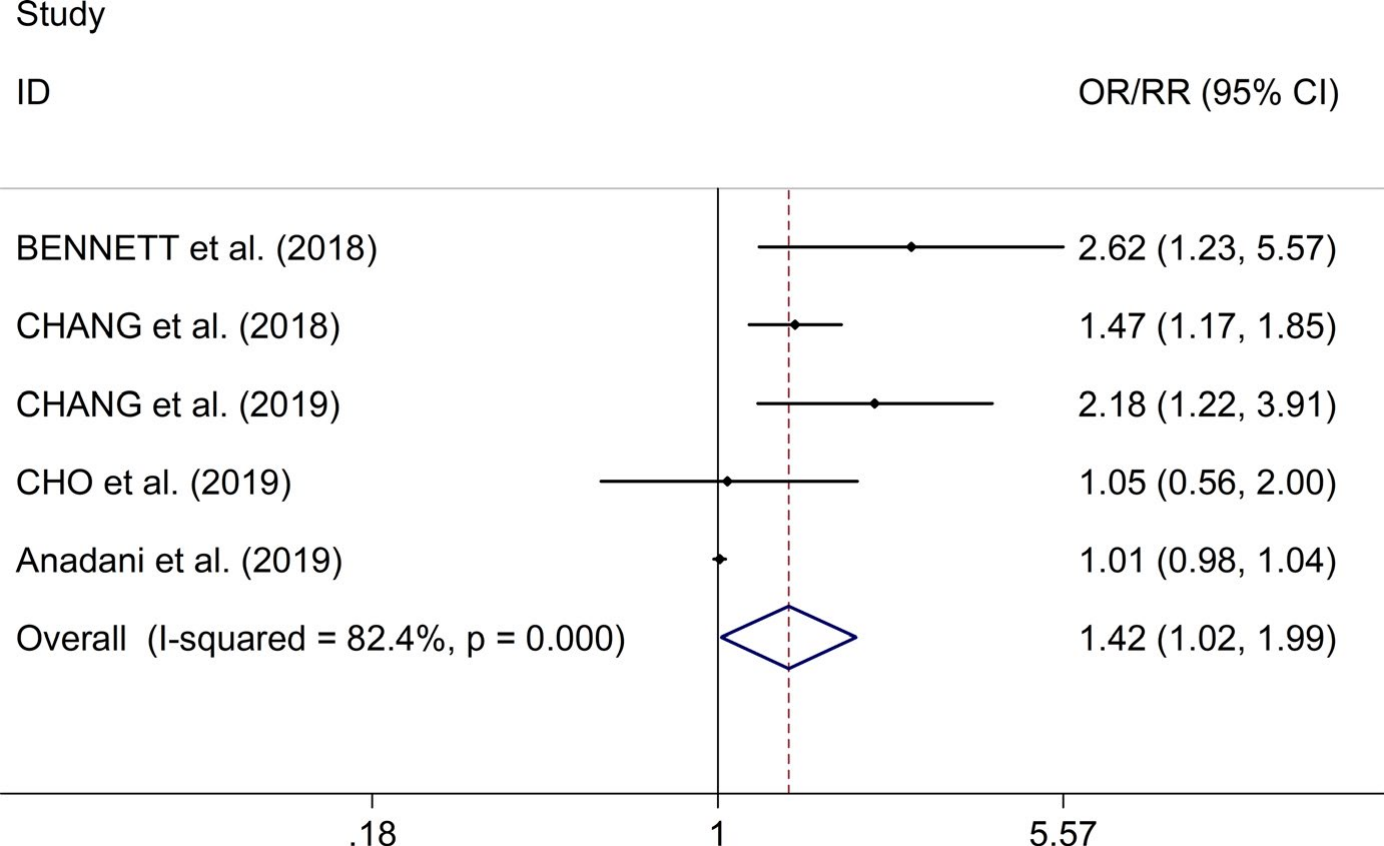
Forest plots for the effect of a defined increment (per 10‐mm Hg increase or 1‐*SD* increase) in either early CV or *SD* of SBP on mRS after EVT in AIS. Abbreviations: AIS, acute ischemic stroke; CI, confidence intervals; CV, coefficient of variation; EVT, endovascular thrombectomy; mRS, modified Rankin Scale; OR, odds ratio; SBP, systolic blood pressure

Regarding the effect of early SBPV after EVT on functional outcome [evaluated with 3‐month modified Rankin Scale (mRS)], the meta‐analysis with fixed effects models showed that increased SBPV after EVT was significantly associated with worse functional outcome in AIS (OR/RR = 1.42, 95% CI 1.02 to 1.99, *I* ^2^ = 82.4%, *p* value of *Q* test < .001, Figure 2). In the study, sensitivity analysis showed no changes in the direction of effect when excluding any one study (Figure S1). Bennett et al. (2018) reported that increased BPV during 24 hr after EVT as measured by the *SD* and CV consistently predict worse functional outcomes as measured by follow‐up mRS in AIS patients. Chang et al. (2018) reported that short‐term BPV over 24 and 48 hr after EVT in AIS patients may be an independent predictor of functional outcome. Chang et al. (2019) reported that BPV during 48 hr after EVT remained significant to predict functional outcomes at 3 months in AIS patients with poor collateral circulation, whereas no significant association was found between BPV parameters and clinical outcomes in patients with good collateral circulation. Cho and Kim (2019) showed no significant association between SBPV during the first 24 hr after EVT and functional outcomes at 3 months in AIS patients. Anadani et al. (2019) reported that no association between SBPV during the first 24 hr after EVT and functional outcomes at 3 months in AIS patients. Additionally, both 2 studies (Anadani et al., 2019; Cho & Kim, 2019) reported that SBPV after EVT was not significantly associated with sICH or mortality at 3 months in AIS.

**FIGURE 3:**
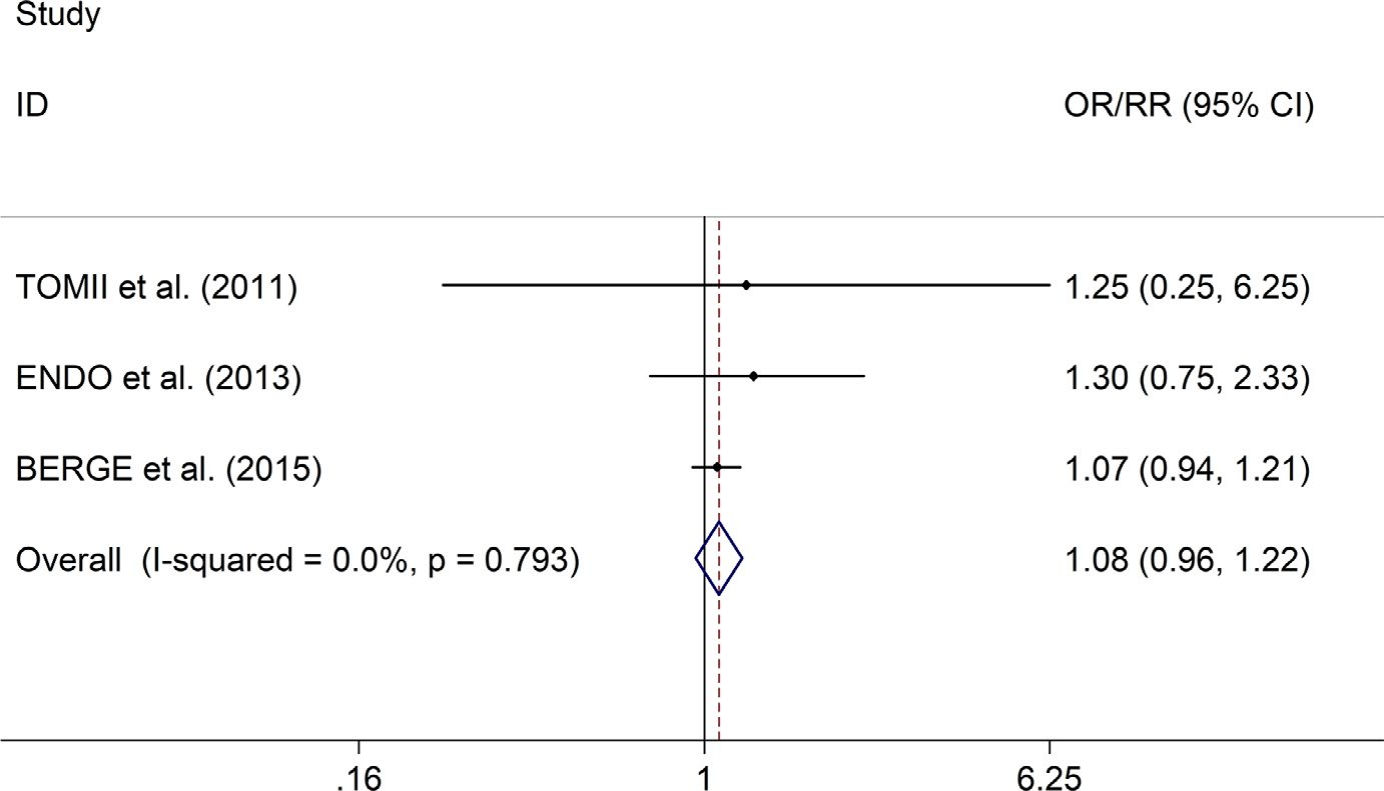
Forest plots for the effect of a defined increment (per 10‐mm Hg increase or 1‐*SD* increase) in either early CV or *SD* of SBP on mRS after IVT in AIS. Abbreviations: AIS, acute ischemic stroke; CI, confidence intervals; CV, coefficient of variation; IVT, intravenous thrombolysis; mRS, modified Rankin Scale; OR, odds ratio; SBP, systolic blood pressure; *SD*, standard deviation

**FIGURE 4:**
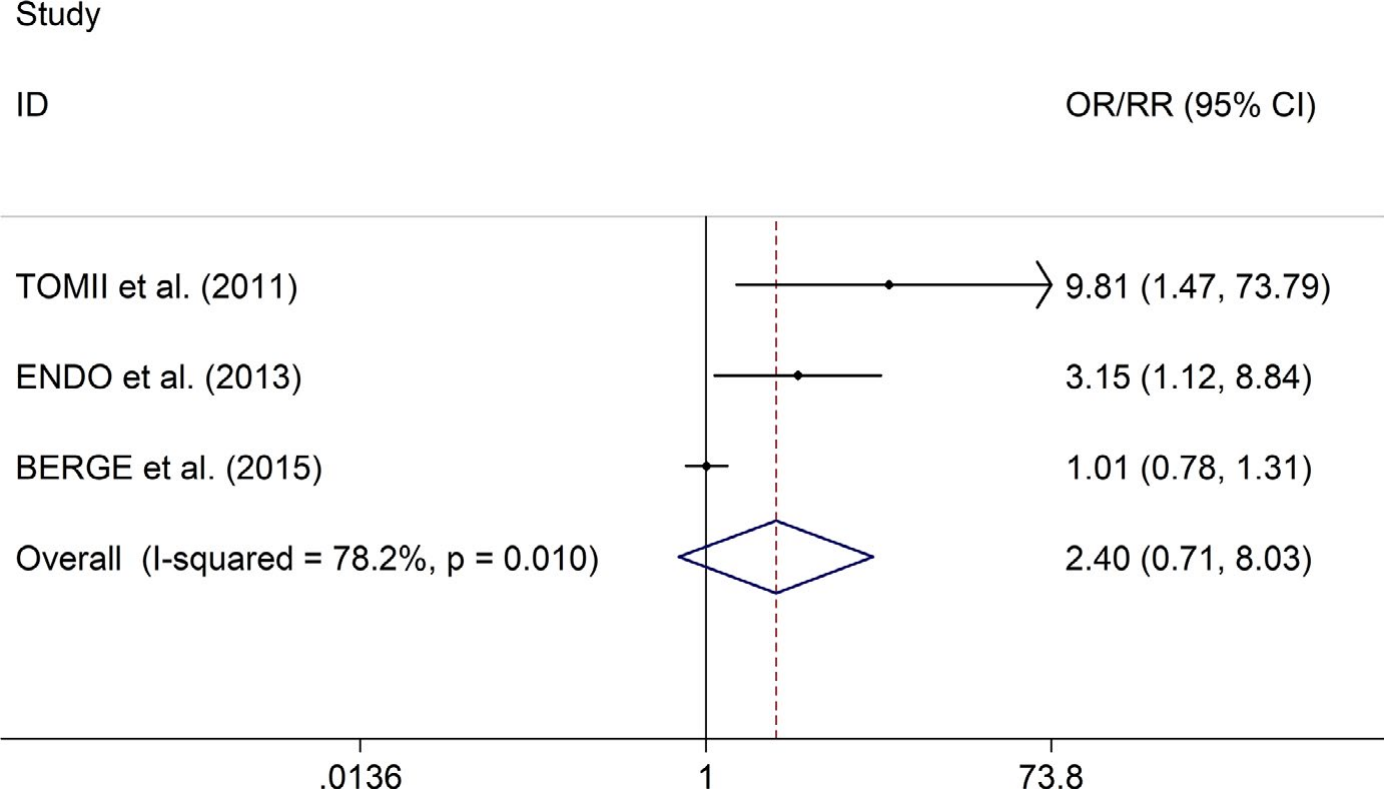
Forest plots for the effect of a defined increment (per 10‐mm Hg increase or 1‐*SD* increase) in either early CV or *SD* of SBP on sICH after IVT in AIS. Abbreviations: AIS, acute ischemic stroke; CI, confidence intervals; CV, coefficient of variation; sICH, symptomatic intracerebral hemorrhage; IVT, intravenous thrombolysis; OR, odds ratio; SBP, systolic blood pressure; *SD*, standard deviation

Regarding the effect of early SBPV after IVT on functional outcome [evaluated with 3‐month or 6‐month mRS], the meta‐analysis with fixed effects models indicated no significant association between SBPV after IVT and functional outcome in AIS (OR/RR = 1.08, 95% CI 0.96–1.22, *I* ^2^ = 0.0%, *p* value of *Q* test = .793, Figure 3). In the study, sensitivity analysis showed no changes in the direction of effect when excluding any one study (Figure S2). Regarding the effect of early SBPV after IVT on sICH, the meta‐analysis with random effects models showed no significant association between SBPV after IVT and sICH in AIS (OR/RR = 2.40, 95% CI 0.71–8.03, *I* ^2^ = 78.2%, *p* value of *Q* test = 0.01, Figure 4). Sensitivity analysis indicated a change in the direction of effect when excluding the study made by Berge et al. (2015) (Figure S3, S1). Tomii et al. (2011) found no significant association between SBPV and functional outcome after IVT, whereas an early SBPV was positively associated with sICH after IVT. Endo et al. (2013) reported no significant association between SBPV and functional outcome, whereas an early SBPV was positively associated with sICH and death after IVT. Berge et al. (2015) indicated no significant associations between early SBPV after IVT and functional outcome and sICH, whereas AIS patients with increased SBPV experienced more early adverse events. Subgroup study further showed that the association between SBPV and functional outcome was affected by onset to treatment time.

The risk of bias graph is shown in Figure S4. Details of the risk of bias summary can be found in Figure S5.

## 4 DISCUSSION

The present study indicated that increased early SBPV after EVT was significantly associated with worse functional outcome in AIS, whereas no significant associations were indicated between SBPV after IVT and functional outcome, sICH in AIS.

The present study showed significant associations between increased early SBPV after EVT and worse functional outcome in AIS. A previous meta‐analysis indicated that increased BPV in AIS might be associated with worse functional outcome (Manning et al., 2015). The present study focused on the association between BPV after EVT and functional outcome in AIS. Increased BPV during the initial few days after AIS was related to poor outcomes, partly because BPV affected cerebral perfusion (Bhatt & Farooq, 2009; Sare et al., 2009). The better functional outcome might be attributed to successful vessel recanalization. Previous studies indicated that successful vessel recanalization can mainly be attributed to SBP decrement after EVT (Mattle et al., 2005). Recent studies showed that lower BPV is also related to better recanalization (Chang et al., 2018, 2019). Additionally, recanalization status might have an influence on the relationship between BP and functional outcomes after either EVT or IVT thrombolysis (Cho & Kim, 2019; Gerschenfeld et al., 2017; Martins et al., 2016). Cho et al. found that higher SBPV was related to worse functional outcomes after EVT in substantially reperfused AIS patients but not in nonreperfused AIS patients (Cho & Kim, 2019). However, a recent study indicated that increased BPV was related to poor outcomes in AIS patients with incomplete reperfusion after EVT (Bennett et al., 2018). Both of the studies showed a significant moderate effect of reperfusion status on the associations between SBPV and functional outcomes. Moreover, Chang et al. reported significant associations between BPV and worse functional outcomes at 3 months in AIS patients with poor collateral circulation after EVT, whereas no significant associations were showed between BPV and clinical outcomes in AIS patients with good collateral circulation (Chang et al., 2019). In summary, the damage severity in cerebral autoregulation might moderate the pathophysiological influence of BPV on brain tissue. Cerebral autoregulation may be affected by infarct size (Guo et al., 2014; Reinhard et al., 2012). Therefore, in those patients with more severe AIS and increased cerebral dysautoregulation, the impact of BPV on pathophysiology was increased compared to those with milder AIS (Laurent et al., 2009; Manning et al., 2015; Sykora et al., 2008). These studies indicated that increased early SBPV after EVT is related to worse functional outcome in AIS, but different BP management strategies on the basis of reperfusion status and collateral circulation after EVT might be needed. Additionally, a previous study (Cho & Kim, 2019) did not report any independent association between SBPV after EVT and sICH or mortality as it was published for IVT (Berge et al., 2015; Endo et al., 2013; Tomii et al., 2011). High BP or BPV might be generally considered to be associated with a high probability of sICH and mortality. However, sICH after EVT is a complex performance and is affected by some clinical factors (Mokin et al., 2012). However, no study explored the possible factors which might affect the incidence of sICH. In addition, some previous studies on EVT showed no significant association between sICH and BP (Goyal et al., 2017; Kellert et al., 2012).

The present study showed no significant associations were indicated between early SBPV after IVT and functional outcome, sICH in AIS. A recent study reported that lower pretreatment and post‐treatment SBP levels were associated with better 3‐month functional outcomes in AIS (Malhotra et al., 2019). The present study focused on the association between BPV after IVT and functional outcome in AIS. Berge et al. (2015) with subgroup study further showed that the association between SBPV and functional outcome was affected by onset to treatment time. Thus, further large‐scale studies are essential to explore the factors affecting the association between early SBPV and functional outcome.

There are some limitations in the present study. First, regarding the effect of early SBPV after IVT on functional outcome and sICH, there were a limited number of studies, potentially limiting statistical power. Second, the limited total number of included studies was limited to do subgroup analyses to explore the source of heterogeneity. Third, the exclusion of studies with insufficient data may lead to a potential bias.

## 5 CONCLUSIONS

In conclusion, these studies provide evidence that increased early SBPV after EVT is related to worse longer‐term functional outcome in AIS, but the association is not significant in AIS patients treated with IVT. In addition, further prospective and large‐scale study is essential to explore the factors affecting the association between early SBPV and functional outcome. Furthermore, individualized BP management strategies were essential for AIS patients after EVT or IVT.

## CONFLICT OF INTERESTS

No conflict of interests.

## AUTHOR CONTRIBUTION

Jingcui Qin participated in research design, the writing of the paper, the performance of the research, and data analysis. Zhijun Zhang participated in research design and data analysis.

### Peer Review

The peer review history for this article is available at https://publons.com/publon/10.1002/brb3.1898.

## Supporting information

Table S1

Figure S1

Figure S2

Figure S3

Figure S4

Figure S5

## Data Availability

The present study was a meta‐analysis. I have attached all data in Table S1.
