# Supplementary material for: Prognostic significance of early systolic blood pressure variability after endovascular thrombectomy and intravenous thrombolysis in acute ischemic stroke: A systematic review and meta‐analysis
Source: Brain Behav. 2020 Oct 14;10(12):e01898. doi: 10.1002/brb3.1898 (PMC7749530; doi:10.1002/brb3.1898)
Supplement: Supplementary file 1 — Table S1 [file BRB3-10-e01898-s001.docx]

Supplementary table 1. Characteristics of all included studies.

| Study/Year | Study type | Country | Patient Numbers | Age | Therapy Given | Average Initial  Stroke Severity | Pre-treatment systolic blood pressure | Antihypertensive treatment | Time From Stroke  Onset to Recruitment | BP Measurement Technique and Duration of  Monitoring | BPV Parameters  Included in Analyses | Outcome Measures |
| --- | --- | --- | --- | --- | --- | --- | --- | --- | --- | --- | --- | --- |
| BENNETT et al. (2018) [^1^](#_ENREF_1) | retrospectively observational analysis | USA | 182 | 63.2 (16.3) | endovascular thrombectmy | NIHSS 16 (10-22) | Hypertension 55.0% | NR | <24 h | NR, 120 h | SD, CV, SV  during 0-24h | Functional outcome |
| CHANG et al. (2018) [^2^](#_ENREF_2) | retrospectively observational analysis | Korea | 303 | 72.0 (64.5-78.0) | Intra-Arterial Thrombectomy | NIHSS 15 (9-20) | NR | NR | <48 h | Casual cuff BP for 48 h | Mean, SD, CV, VIM | Functional outcome |
| CHANG et al. (2019) [^3^](#_ENREF_3) | retrospectively observational analysis | Korea | 90 | 72.3 (11.8) | Endovascular  Thrombectomy | NIHSS 14.7 (5.5) | 149.0  (134.0-166.0) | NR | <48 h | Casual cuff BP for 48 h | Mean, SD, CV, VIM | Functional outcome |
| CHO et al. (2019) [^4^](#_ENREF_4) | retrospectively observational analysis | Korea | 378 | 70.0 (11.4) | endovascular thrombectmy | NIHSS 12 (9- 15) | Hypertension 58.7% | NR | <24 h | NR | Initial, mean, max, SD, CV, VIM, SV | Functional outcome,sICH or mortality |
| Anadani et al. (2019) [^5^](#_ENREF_5) | retrospectively observational analysis | USA | 1245 | 69 (14) | endovascular thrombectmy | NR | Hypertension 64% | Yes, as per normal | <24 h | a BP cuff or an arterial line for 24 h | SD |  |
| TOMII et al. (2011) [^6^](#_ENREF_6) | Prospectively observational analysis | Japan | 125 | 72.7(9.0) | Intravenous thrombolysis with rt-PA | NIHSS 13 (7-18) | Hypertension 71% | Yes, as per normal  clinical practice | <3 h | Casual cuff for 24 h | CV | Functional outcome,  sICH |
| ENDO et al. (2013) [^7^](#_ENREF_7) | Observational analysis (stroke registry) | Japan | 527 | NR | Intravenous  Thrombolysis rt-PA 0.6 mg/kg | NIHSS 12 (7-18) | NR | Yes, as per normal  clinical practice | <6 h | Casual cuff BP for 24 h | SD, SV, CV | Functional outcome,  sICH |
| BERGE et al. (2015) [^8^](#_ENREF_8) | prospectively observational analysis | Australia | 3035 | 81 (72-86) | Intravenous  Thrombolysis rt-PA 0.9 mg/kg | NIHSS≤10: n = 1461; >10: n = 1567 | NR | Yes, as per normal  clinical practice | <6 h | Casual cuff BP for 24 h | SD | Functional outcome,  sICH |

Abbreviations: BP, blood pressure; BPV, blood pressure variability; CV, coefficient of variation; sICH, symptomatic intracerebral hemorrhage; MRI, magnetic resonance imaging; NIHSS, National Institute of Health Stroke Scale; NR, not reported; RCT, randomized controlled trial; rt-PA, recombinant tissue plasminogen activator; SBP, systolic blood pressure; SD, standard deviation; SV, successive variation; USA, united states; VIM, variation independent of the mean.

Supplementary figure 1. Sensitivity analysis for the effect of a defined increment (per 10-mm Hg increase or 1-SD increase) in either early CV of SBP on mRS after EVT in AIS. Abbreviations: AIS, acute ischemic stroke; CV, coefficient of variation; EVT, endovascular thrombectomy; mRS, modified Rankin Scale; SBP, systolic blood pressure.

**
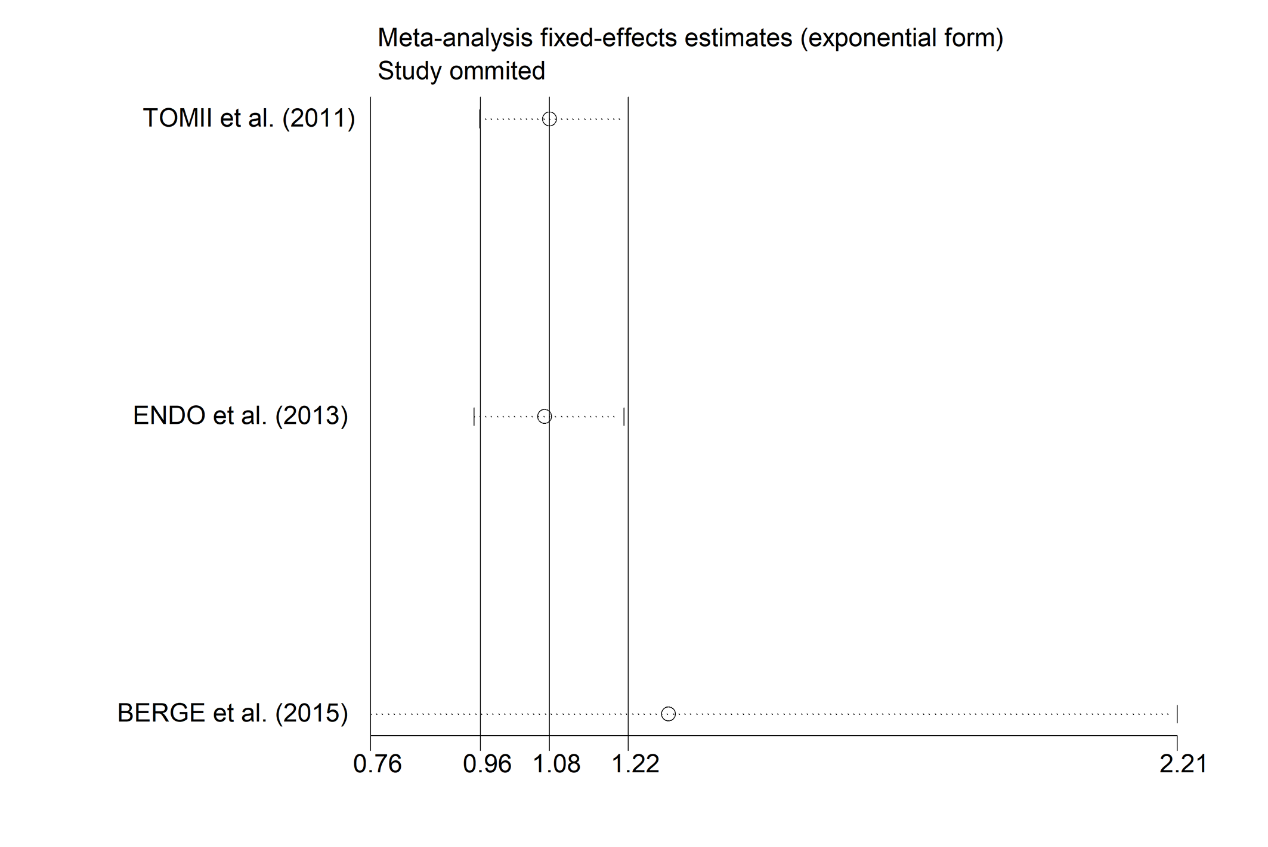
**

Supplementary figure 2. Sensitivity analysis for the effect of a defined increment (per 10-mm Hg increase or 1-SD increase) in either early CV or SD of SBP on mRS after IVT in AIS. Abbreviations: AIS, acute ischemic stroke; CV, coefficient of variation; IVT, intravenous thrombolysis; mRS, modified Rankin Scale; SBP, systolic blood pressure; SD, standard deviation.


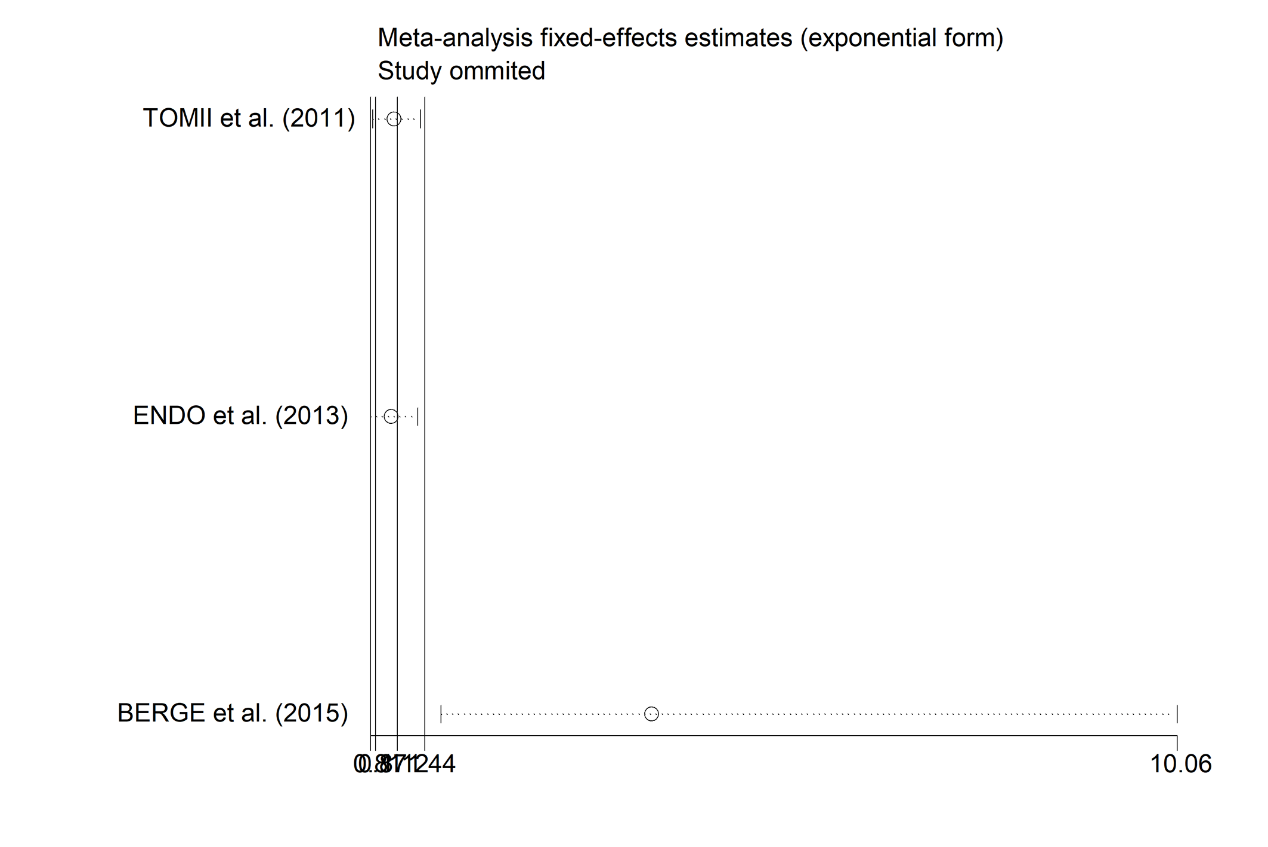


Supplementary figure 3. Sensitivity analysis for the effect of a defined increment (per 10-mm Hg increase or 1-SD increase) in either early CV or SD of SBP on ICH after IVT in AIS. Abbreviations: AIS, acute ischemic stroke; CV, coefficient of variation; ICH, intracerebral hemorrhage; IVT, intravenous thrombolysis; SBP, systolic blood pressure; SD, standard deviation.


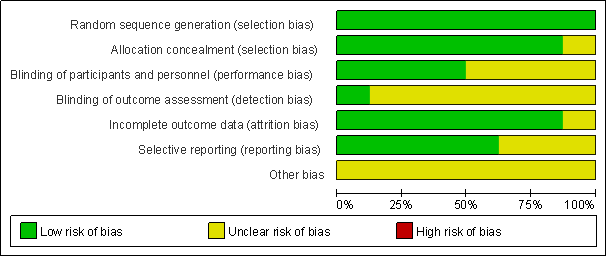


Supplementary figure 4. Risk of bias graph.


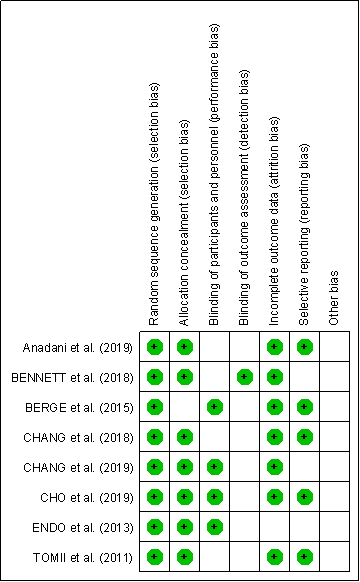


Supplementary figure 5. Details of the risk of bias summary.

**Supplementary references:**

1. Bennett AE, Wilder MJ, McNally JS, et al. Increased blood pressure variability after endovascular thrombectomy for acute stroke is associated with worse clinical outcome. Journal of neurointerventional surgery. 2018;10(9):823-827.

2. Chang JY, Jeon SB, Lee JH, Kwon OK, Han MK. The Relationship between Blood Pressure Variability, Recanalization Degree, and Clinical Outcome in Large Vessel Occlusive Stroke after an Intra-Arterial Thrombectomy. Cerebrovascular diseases (Basel, Switzerland). 2018;46(5-6):279-286.

3. Cho BH, Kim JT. Associations of various blood pressure parameters with functional outcomes after endovascular thrombectomy in acute ischaemic stroke. 2019;26(7):1019-1027.

4. Anadani M, Orabi MY, Alawieh A, et al. Blood Pressure and Outcome After Mechanical Thrombectomy With Successful Revascularization. Stroke. 2019;50(9):2448-2454.

5. Tomii Y, Toyoda K, Nakashima T, et al. Effects of hyperacute blood pressure and heart rate on stroke outcomes after intravenous tissue plasminogen activator. Journal of hypertension. 2011;29(10):1980-1987.

6. Endo K, Kario K, Koga M, et al. Impact of early blood pressure variability on stroke outcomes after thrombolysis: the SAMURAI rt-PA Registry. Stroke. 2013;44(3):816-818.

7. Berge E, Cohen G, Lindley RI, et al. Effects of Blood Pressure and Blood Pressure-Lowering Treatment During the First 24 Hours Among Patients in the Third International Stroke Trial of Thrombolytic Treatment for Acute Ischemic Stroke. Stroke. 2015;46(12):3362-3369.

| **Section/topic** | **#** | **Checklist item** | **Reported on page #** |
| --- | --- | --- | --- |
| **TITLE** | | |  |
| Title | 1 | Identify the report as a systematic review, meta-analysis, or both. | Title page |
| **ABSTRACT** | | |  |
| Structured summary | 2 | Provide a structured summary including, as applicable: background; objectives; data sources; study eligibility criteria, participants, and interventions; study appraisal and synthesis methods; results; limitations; conclusions and implications of key findings; systematic review registration number. | 1 |
| **INTRODUCTION** | | |  |
| Rationale | 3 | Describe the rationale for the review in the context of what is already known. | 1, 2 |
| Objectives | 4 | Provide an explicit statement of questions being addressed with reference to participants, interventions, comparisons, outcomes, and study design (PICOS). | 2 |
| **METHODS** | | |  |
| Protocol and registration | 5 | Indicate if a review protocol exists, if and where it can be accessed (e.g., Web address), and, if available, provide registration information including registration number. | 2 |
| Eligibility criteria | 6 | Specify study characteristics (e.g., PICOS, length of follow-up) and report characteristics (e.g., years considered, language, publication status) used as criteria for eligibility, giving rationale. | 2 |
| Information sources | 7 | Describe all information sources (e.g., databases with dates of coverage, contact with study authors to identify additional studies) in the search and date last searched. | 2 |
| Search | 8 | Present full electronic search strategy for at least one database, including any limits used, such that it could be repeated. | 2 |
| Study selection | 9 | State the process for selecting studies (i.e., screening, eligibility, included in systematic review, and, if applicable, included in the meta-analysis). | 2 |
| Data collection process | 10 | Describe method of data extraction from reports (e.g., piloted forms, independently, in duplicate) and any processes for obtaining and confirming data from investigators. | 2 |
| Data items | 11 | List and define all variables for which data were sought (e.g., PICOS, funding sources) and any assumptions and simplifications made. | 2 |
| Risk of bias in individual studies | 12 | Describe methods used for assessing risk of bias of individual studies (including specification of whether this was done at the study or outcome level), and how this information is to be used in any data synthesis. | 2, 3 |
| Summary measures | 13 | State the principal summary measures (e.g., risk ratio, difference in means). | 2, 3 |
| Synthesis of results | 14 | Describe the methods of handling data and combining results of studies, if done, including measures of consistency (e.g., I^2^) for each meta-analysis. | 2, 3 |

| Risk of bias across studies | 15 | Specify any assessment of risk of bias that may affect the cumulative evidence (e.g., publication bias, selective reporting within studies). | 3 |
| --- | --- | --- | --- |
| Additional analyses | 16 | Describe methods of additional analyses (e.g., sensitivity or subgroup analyses, meta-regression), if done, indicating which were pre-specified. | 3 |
| **RESULTS** | | |  |
| Study selection | 17 | Give numbers of studies screened, assessed for eligibility, and included in the review, with reasons for exclusions at each stage, ideally with a flow diagram. | 3 |
| Study characteristics | 18 | For each study, present characteristics for which data were extracted (e.g., study size, PICOS, follow-up period) and provide the citations. | 3 |
| Risk of bias within studies | 19 | Present data on risk of bias of each study and, if available, any outcome level assessment (see item 12). | 3, 4 |
| Results of individual studies | 20 | For all outcomes considered (benefits or harms), present, for each study: (a) simple summary data for each intervention group (b) effect estimates and confidence intervals, ideally with a forest plot. | 3, 4 |
| Synthesis of results | 21 | Present results of each meta-analysis done, including confidence intervals and measures of consistency. | 3, 4 |
| Risk of bias across studies | 22 | Present results of any assessment of risk of bias across studies (see Item 15). | 3, 4 |
| Additional analysis | 23 | Give results of additional analyses, if done (e.g., sensitivity or subgroup analyses, meta-regression [see Item 16]). | 3, 4 |
| **DISCUSSION** | | |  |
| Summary of evidence | 24 | Summarize the main findings including the strength of evidence for each main outcome; consider their relevance to key groups (e.g., healthcare providers, users, and policy makers). | 4 |
| Limitations | 25 | Discuss limitations at study and outcome level (e.g., risk of bias), and at review-level (e.g., incomplete retrieval of identified research, reporting bias). | 5 |
| Conclusions | 26 | Provide a general interpretation of the results in the context of other evidence, and implications for future research. | 5 |
| **FUNDING** | | |  |
| Funding | 27 | Describe sources of funding for the systematic review and other support (e.g., supply of data); role of funders for the systematic review. | 5 |

*From:*  Moher D, Liberati A, Tetzlaff J, Altman DG, The PRISMA Group (2009). Preferred Reporting Items for Systematic Reviews and Meta-Analyses: The PRISMA Statement. PLoS Med 6(6): e1000097. doi:10.1371/journal.pmed1000097

For more information, visit: **www.prisma-statement.org**.

Page 2 of 2
