# Supplementary figures and images for: Prognostic significance of early systolic blood pressure variability after endovascular thrombectomy and intravenous thrombolysis in acute ischemic stroke: A systematic review and meta‐analysis
Source: Brain Behav. 2020 Oct 14;10(12):e01898. doi: 10.1002/brb3.1898 (PMC7749530; doi:10.1002/brb3.1898)

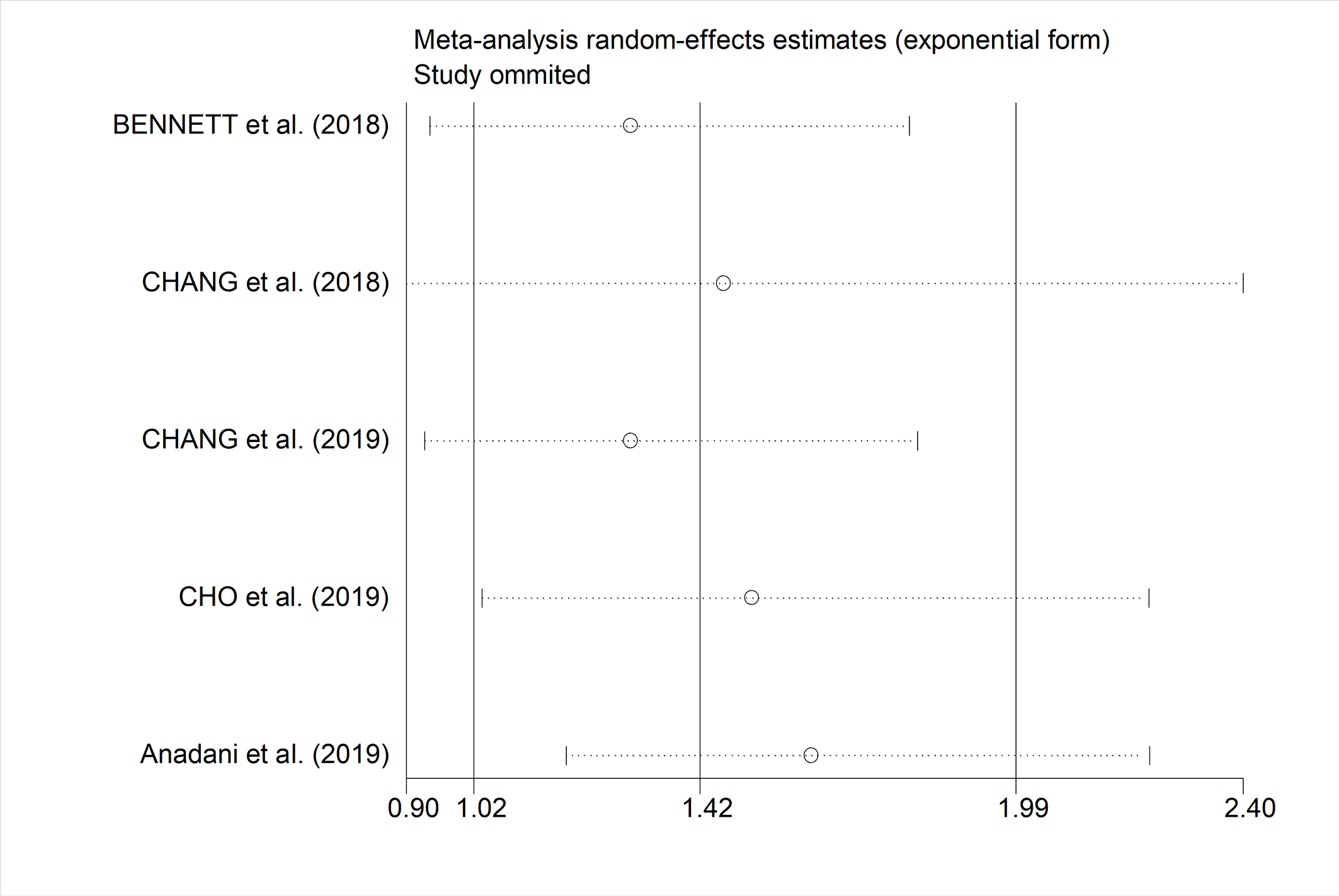

Supplement: Supplementary file 2 — Figure S1 [file BRB3-10-e01898-s002.tif]

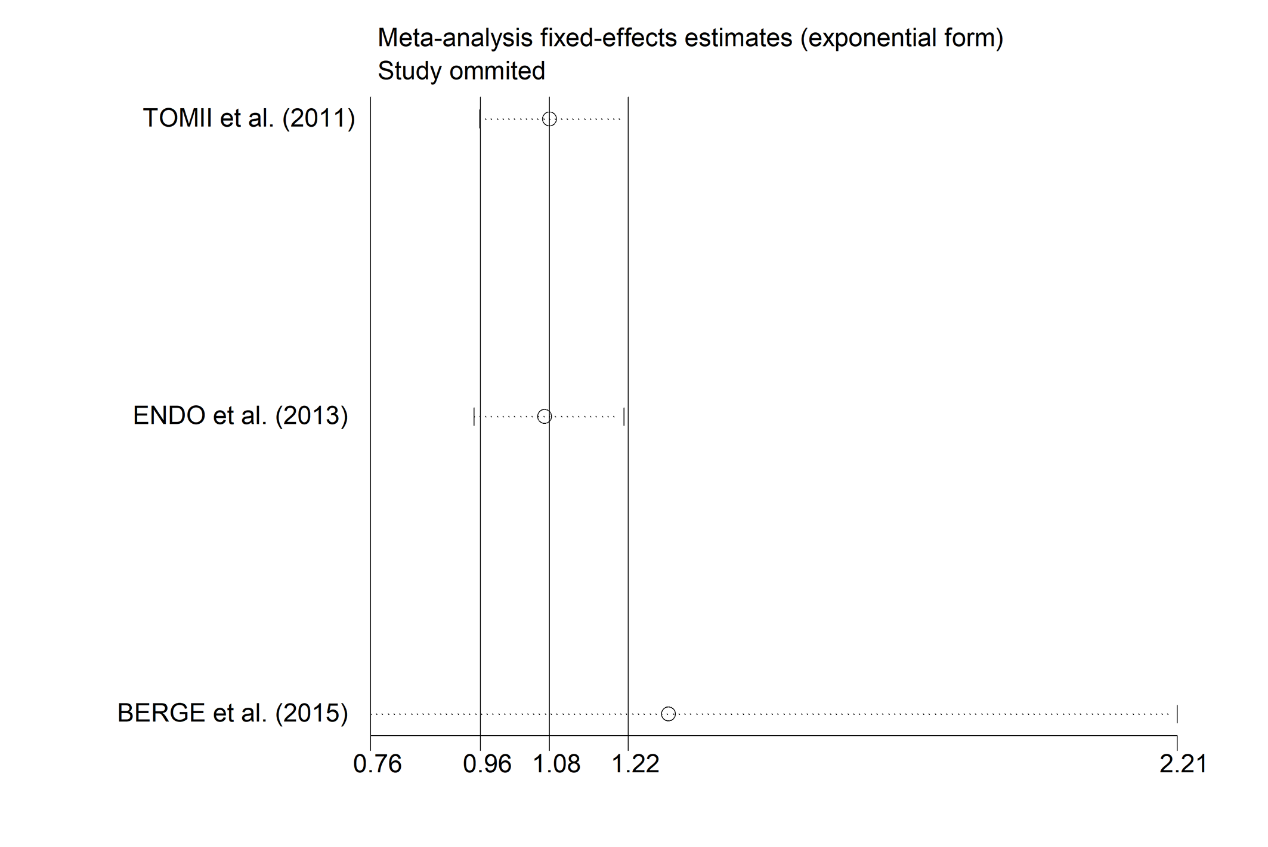

Supplement: Supplementary file 3 — Figure S2 [file BRB3-10-e01898-s003.tif]

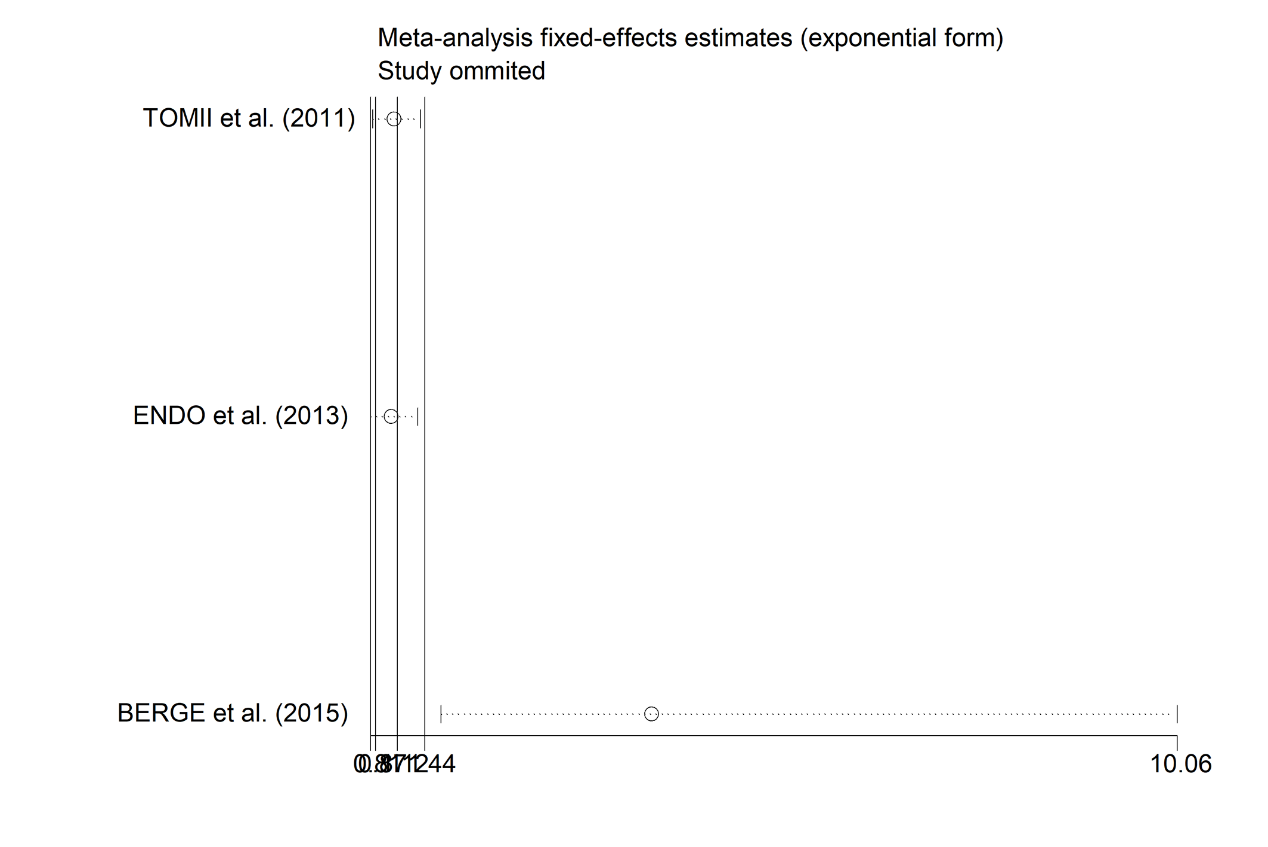

Supplement: Supplementary file 4 — Figure S3 [file BRB3-10-e01898-s004.tif]

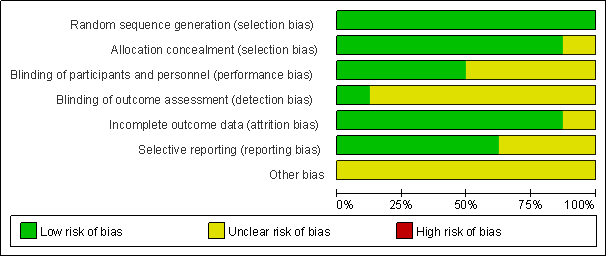

Supplement: Supplementary file 5 — Figure S4 [file BRB3-10-e01898-s005.tif]

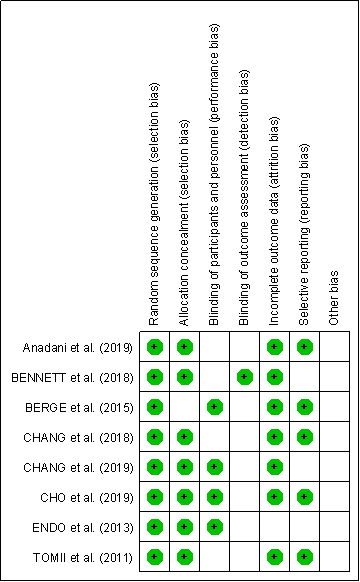

Supplement: Supplementary file 6 — Figure S5 [file BRB3-10-e01898-s006.tif]
